# A Systematic Review of 4D-Flow MRI Derived Mitral Regurgitation Quantification Methods

**DOI:** 10.3389/fcvm.2019.00103

**Published:** 2019-08-02

**Authors:** Benjamin Fidock, Natasha Barker, Nithin Balasubramanian, Gareth Archer, Graham Fent, Abdullah Al-Mohammad, James Richardson, Laurence O'Toole, Norman Briffa, Alexander Rothman, Rob van der Geest, Rod Hose, James M. Wild, Andrew J. Swift, Pankaj Garg

**Affiliations:** ^1^Department of Infection, Immunity & Cardiovascular Disease, University of Sheffield, Sheffield, United Kingdom; ^2^Sheffield Teaching Hospitals NHS Foundation Trust, Sheffield, United Kingdom; ^3^Leiden University Medical Centre, Leiden, Netherlands

**Keywords:** mitral regurgitation, 4D flow CMR, 4D flow MRI, phase contrast, echocardiography, retrospective-valve-tracking

## Abstract

**Background:** Four-dimensional flow cardiac magnetic resonance (4D flow CMR) is an emerging non-invasive imaging technology that can be used to quantify mitral regurgitation (MR) volume. Current methods of quantification have demonstrated limitations in accurate analysis, particularly in difficult cases such as complex congenital heart disease. 4D flow CMR methods aim to circumvent these limitations and allow accurate quantification of MR volume even in complex cases. This systematic review aims to summarize the available literature on 4D flow CMR MR quantification methods and examine their ability to accurately classify MR severity.

**Methods:** Structured searches were carried out on Medline and EMBASE in December 2018 to identify suitable research outcome studies. The titles and abstracts were screened for relevance, with a third adjudicator utilized when study suitability was uncertain.

**Results:** Seven studies met the eligibility criteria and were included in the systematic review. The most widely used 4D flow MRI method was retrospective valve tracking (RVT) which was examined in five papers. The key finding of these papers was that RVT is a reliable and accurate method of regurgitant volume quantification.

**Conclusions:** MR quantification through 4D flow MRI is both feasible and accurate. The evidence gathered suggests that for MR assessment, 4D flow MRI is potentially as accurate and reliable to echocardiography and may be complementary to this technique. Further work on MR quantification 4D flow image analysis is needed to determine the most accurate analysis technique and to demonstrate 4D flow MRI as a predictor of clinical outcome.

**PROSPERO Registration Number:** CRD42019122837, http://www.crd.york.ac.uk/PROSPERO/display_record.php?ID=CRD42019122837

## Introduction

Mitral regurgitation (MR) is a serious disease of the mitral valve where blood is allowed to flow from the left ventricle to the left atrium during systole. Primary MR is caused by a defect in at least one aspect of the valve apparatus (e.g., mitral valve leaflet prolapse), whereas secondary MR is a consequence of other cardiac disease involving the myocardium (e.g., dilated cardiomyopathy, myocardial infarction) (1). MR is the second most common valvular condition and is particularly prevalent in elderly populations; affecting ~9% of over 75 s (2). Patients with MR can live for many years without symptoms and then suddenly decompensate with acute heart failure. Mild or moderate MR is often conservatively managed. Severe MR is most commonly treated through surgical repair or replacement of the valve, although transcatheter intervention techniques are becoming more frequent (3). The clinical management of MR is based on many factors including left ventricular size, left ventricular function and patient symptoms. Current ESC guidelines place emphasis on the left ventricular ejection fraction (LVEF) and left ventricular end-diastolic volume (LVEDV) as indicators for surgery. A LVEF ≤ 60% or LVESD ≥ 45 mm are triggers for surgery in asymptomatic patients. In addition, surgery should be considered in patients with atrial fibrillation secondary to MR or pulmonary hypertension. Patient assessment can be made more difficult by confounding symptoms from commonly associated diseases or the lack of symptoms even in cases of severe MR (1, 4, 5). The timing of intervention is often reliant on the accurate quantification of MR severity; this is crucial to prevent increased morbidity and mortality in this patient group. Larger regurgitant volumes have a strong correlation with increasing mortality, no matter the background pathophysiology (3, 6). It is therefore important to accurately and precisely quantify MR to guide clinical decisions.

In routine clinical practice, echocardiography is the standard assessment tool for MR. However, a limited number of recent studies have shown that cardiac magnetic resonance (CMR) to be more accurate when quantifying organic MR and superior at predicting clinical outcomes when compared to echocardiography. CMR is the reference imaging modality for the assessment of left ventricular volumes and ejection fractions. In addition, it allows clinicians to make an assessment of the mitral valve morphology and classify MR as primary or secondary. The addition of late gadolinium enhancement to standard MRI protocol enables the evaluation of localized myocardial fibrosis which can be a result of chronic MR. The extent of fibrosis has been shown to independently predict outcomes (7). Therefore, CMR is a versatile non-invasive tool that allows comprehensive multi-parametric assessment of MR and its etiology (8).

MR can be quantified either directly or indirectly by both CMR and echocardiography. Both indirect methods calculate the difference between left ventricular stroke volume (LVSV) and aortic forward volume (AoPC). This is the current standard, and most widely used, method of CMR and flow quantification can be obtained through phase contrast (PC) velocity encoded imaging. This technique enables the quantification of regurgitant volume without the consideration of jet number or eccentricity. Alternative methods of quantification can be performed by both proximal isovelocity surface area (PISA) by echocardiography (an indirect method) or by 4-dimensional (4D) flow CMR (a direct method).

Despite the promise of CMR imaging technique there are several limitations that prevent clinical application in all cases. Current CMR MR quantification methods involve two separate types of acquisition and segmentation, which can introduce significant bias and lower limits of agreements between two assessments.

**Figure 1:**
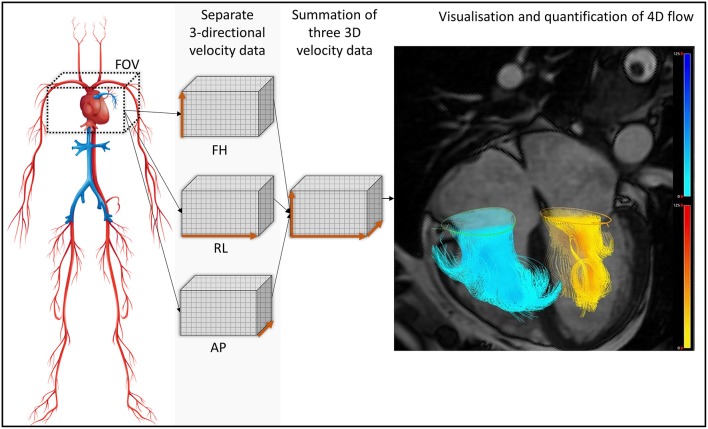
For whole heart 4D flow acquisition, the field of view is planned to cover all chambers of the heart. The bigger the FOV, the longer will be the acquisition and more data will be acquired. Focused studies can be done to save time and circumvent acquisition issues like higher heart rate. Separate three-directional, cross-sectional phase contrast velocity encoded data is acquired throughout the cardiac cycle (30 phases). This is then reconstructed into one cross-sectional velocity data in software packages for analysis. FOV, field of view; FH, foot head direction; RL, right left direction; AP. Anterior posterior direction; 3D, three-dimensional; 4D, four-dimensional (fourth dimension: time).

Researchers aiming to address these problems with current CMR methods have shown significant interest in novel 4-dimensional (4D) flow imaging techniques. 4D flow MRI involves PC acquisition with velocity encoding in three-directions which is also three-dimensional and time-resolved (the fourth dimension; Figure 1). This allows the dynamic visualization of flow in multiple orientations retrospectively. The aim of this systematic review is to methodically summarize 4D flow CMR MR quantification methods and investigate the robustness of these methods in the published literature.

## Methods

### Systematic Review Registration

At inception, this systematic review was prospectively registered (CRD42019122837) with the international prospective register of systematic review (PROSPERO), which is an international database of prospectively registered systematic reviews in health, where there is a health related outcome.

### Eligibility Criteria

Studies which used 4D flow CMR quantification for the assessment of MR were included. We limited our search to peer-reviewed journals, medicine, and human participants. Studies with fewer than 10 patients or those not published in English were excluded.

### Search Strategy

**Figure 2:**
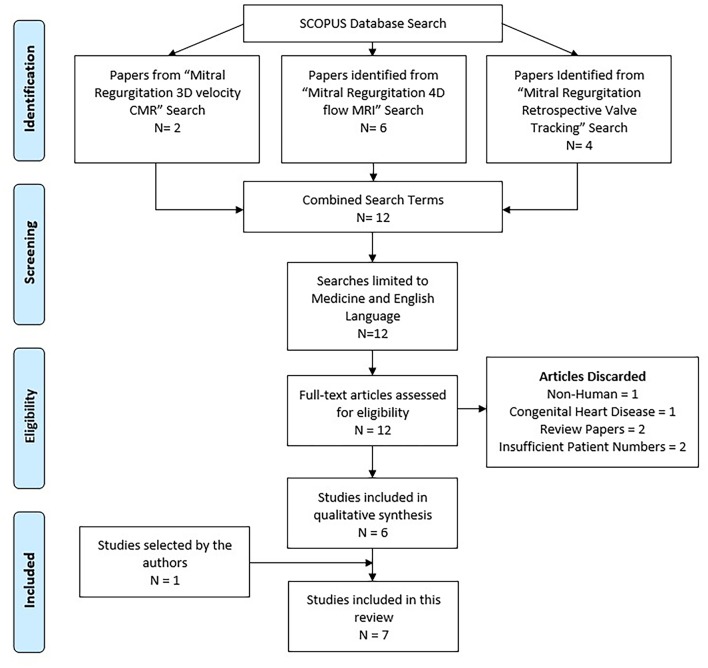
Literature search flow diagram. Adapted from PRISMA tool (10).

The literature search was done on the Scopus database. Scopus is able to undertake 100% search coverage on Medline and EMBASE (9). The search strategy included: *mitral regurgitation 3D velocity CMR* (2 results); *mitral regurgitation 4D flow MRI* (6 results); *mitral regurgitation retrospective valve tracking* (4 results). All searches were combined, and duplicates removed. The search was carried out on 27/11/2018. The flow diagram below demonstrates the literature search based on the preferred reporting items for systematic reviews and meta-analyses (PRISMA; Figure 2).

### Study Selection

PRISMA guidelines were used for study identification, screening and inclusion. Review of the proposed papers was conducted by PG (10-years of cardiology experience) and BF (3-years as a medical student). If there was disagreement, a third reviewer, GA (6-years of cardiology experience), was involved as a deciding vote. To identify any papers missed by the Scopus database, an independent search was carried out with the above search terms on PubMed. After a comprehensive search of the referenced papers, no other relevant papers were identified.

## Results

After a comprehensive review of the content, 7 research outcome studies were identified (6 from Scopus and 1 from PubMed) and included in the systematic review process (Table 1). Papers were excluded for the following reasons: not performed in humans (1), focused on congenital heart disease (1), were review papers (2), or had insufficient patient numbers (2). All 7 papers were electronic sources available online. A summary of these papers can be found in Table 1.

**Table 1:** Summary of all studies included in this systematic review.

**First author year (Place)**	***N***	**Methods of quantification**	**Reproducibility data**	**Comparison**			**Main finding**
				**TTE**	**TOE**	**2D PC MRI**	
Westenberg et al. (Leiden) (10)	30	RVT	–	–	–	+	2D PC MRI overestimated mitral valve flow by 15% in both healthy and MR patients when compared to 4D flow MRI
Marsan et al. (Leiden) (11)	64	RVT	–	+	–	–	4D flow has excellent correlation with RT3DE whilst TTE significantly underestimates regurgitant volume
Roes et al. (Leiden) (12)	51	RVT	+	–	–	–	Intraobserver and interobserver agreements for 4D flow MV volumes of 0.86 and 0.85 respectively
Brandts et al. (Leiden) (13)	47	RVT	–	+	–	+	4D flow found transmitral flow rate to be lower than 2D PC MRI. There is excellent agreement between 2D TTE and 4D flow
Gorodisky et al. (Haifa) (14)	27	CMR - PISA	+	+	–	+	An eccentric shape was found in almost all cases by CMR disagreeing with the hemispheric assumption made in echo PISA
Feneis et al. (San Diego) (15)	21	Direct jet analysis using 4D flow	+	–	–	+	High concordance between 4D flow and 2D PC MRI for both direct and indirect quantification. Good 4D flow interobserver and intraobserver reliability
Kamphuis et al. (Leiden) (16)	160	RVT	+	–	–	–	Automated valve tracking reduces analysis times without an increase in variability

**Figure 3:**
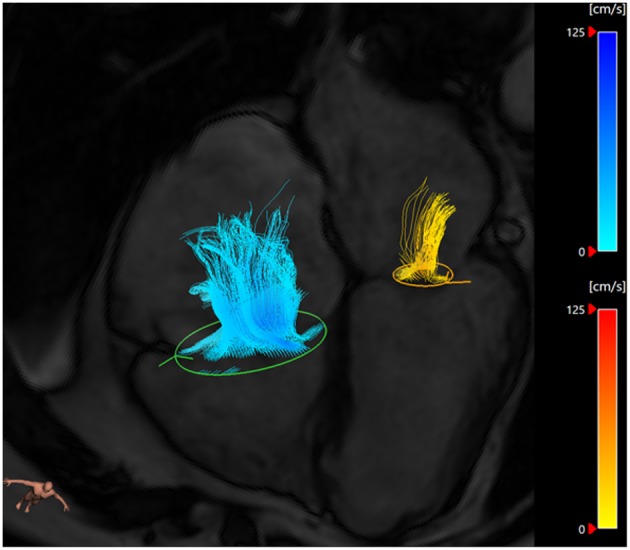
Three-dimensional streamlines visualization of MR (yellow) and tricuspid regurgitation (blue) using retrospective valve tracking on CAAS software (Pie Medical Imaging, Maastricht, The Netherlands). In this case, the MR is mild and there is significant tricuspid regurgitation.

In a 2008 paper, Westenberg et al. directly compared 2D PC MRI to 4D flow CMR in order to validate this technique in the quantification of regurgitation volume. Ten healthy volunteers and 20 patients with either MR, tricuspid regurgitation or both were recruited. The 2D PC MRI overestimated mitral flow by 15% in both healthy and regurgitation patients. This was also seen in this group's future work (13) where 4D CMR was systematically lower than 2D MRI when assessing transmitral flow rate. 4D imaging did not overestimate the flow as it was able to adapt to heart motion and retrospectively track the valve throughout the cardiac cycle (Figure 3).

Brandts et al. (13), compared 4D flow CMR with RVT and 2D TTE Doppler echocardiography for assessing left ventricular filling patterns and calculating regurgitation fractions. They found excellent agreement between the two modalities (k-agreement = 0.91).

Marsan et al. (11), directly compared 4D flow CMR with real-time 3D echocardiography (RT3DE) for quantifying MR. This study included 64 patients with functional MR. Regurgitant volume had little difference between 3D TTE technique vs. 4D flow CMR retrospective valve tracking (RVT) method (mean difference = −0.08 ml/beat). The authors suggested that the small difference between 4D flow CMR and RT3DE may be explained by the fixed position and the resultant misalignment to the flow direction of the RT3DE. Another suggestion was that interference from thoracic obstacles may reduce the accuracy of 3D echocardiography. In addition to comparing 4D flow CMR and RT3DE, a comparison of 2D and 3D echocardiography was conducted. Here the authors observed that 2D echocardiographic methods of MR quantification remain significantly inferior to 3D echocardiographic methods, systematically underestimating regurgitant volume (−2.9 ml/beat, *p* < 0.05). Bland-Altman analysis of the effective regurgitant orifice area in RT3DE showed a mean intraobserver bias (±2D) of 0.04 ±0.04 cm^2^ (*p* = 0.55) and a mean interobserver bias (±2D) of 0.06 ±0.4 cm^2^ (*p* = 0.43). Other papers have found that traditional methods of TTE MR quantification are only modestly reliable and, in many cases, suboptimal (17).

Roes et al. validated 4D flow for flow assessment with all 4 valves simultaneously using retrospective valve tracking. This was performed for both healthy volunteers and patients with valvular regurgitation. For regurgitation fraction Roes et al. found an ICC of 0.86 for intraobserver and 0.85 for interobserver variation. However, this is a collective statistic for all 4 heart valves simultaneously, and not solely for MR.

In 2017 Gorodisky et al. published a paper comparing 2D color Doppler TTE with 3-dimensional proximal flow convergence method of CMR in quantifying MR. The method used enables the true 3D shape of the PISA to be derived from the use of 3D velocity gradients. An eccentric shape was found in almost all cases with a mean eccentricity of 0.81 ± 0.04. The intraclass correlation coefficient (ICC) of their 4D flow method for both experienced and inexperienced users was 0.99. Analysis of the source of bias revealed that this was largely due to the inexperienced user drawing the region of interest marginally too large.

**Figure 4:**
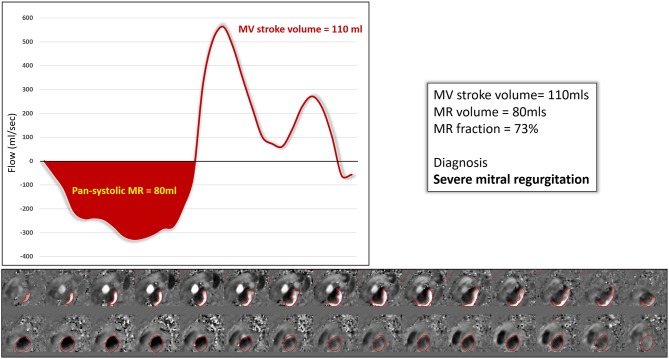
Direct quantification of an eccentric MR jet using 4D flow MRI data. The mitral valve stroke volume in this case was 110 mls and the regurgitation 80 mls. The MR fraction was therefore 73% and the net forward flow 5 mls.

In a recent study done by Feneis et al., the investigators compared 2D PC MRI to 4D flow CMR methods of MR quantification. They measured the inter technique agreement for quantifying MR through direct PC flow measurements (Figure 4) and indirect (LVSV-AoPC) 4D flow analysis and 2D PC. Their results showed that 2D PC and 4D flow yielded equivalent results (Pearson correlation 0.902 and 0.819, respectively). Feneis et al found that interobserver reliability for 4D flow derived MR ranged from very good to excellent for both direct and indirect quantification (*r* = 0.929 and *r* = 0.877, respectively). For direct quantification intraobserver analysis of MR flow volume was excellent for both observers (*r* = 0.988 and *r* = 0.986).

In 2018 Kamphuis et al. published a study comparing automated and manual valve tracking examining; analysis times, net forward volumes and regurgitant fractions. It was found that automated valve tracking analysis times were considerably less than manual times without an increase in variability (14 min [IQR 12–15 min] vs. 25 min [IQR 20–25 min], *P* < 0.001). Interestingly, there was disagreement between the two methods in classification of MR, with the automated method producing 22 discordant cases for MR (k = 0.38, SE = 0.10). Of these 22 cases, 12 cases would have been classified at least mild-to-moderate regurgitation, potentially changing clinical decisions and treatment regimens (1). This study demonstrated that intra- and interobserver variability was improved and variability reduced when automated valve tracking was compared to manual (intraobserver, ICC ≥ 0.99, COV ≤ 5.2%; interobserver, ICC ≥ 0.99, COV ≤ 5.6%). This was seen both in MR patients and healthy volunteers.

## Discussion

The main finding of the evidence synthesized from this systematic review is that 4D flow derived MR quantification methods are reproducible and circumvent issues with standard imaging by both Echocardiography and CMR. As per the current evidence, quantification of MR is best done by direct jet quantification using retrospective valve tracking method on 4D flow MRI. The direct jet quantification by RVT is not only comparable for its accuracy to current standard methods but also demonstrates greater precision in the papers reviewed.

### Comparison Studies to Echocardiography

Echocardiography has long been the standard imaging modality for assessing MR. It is non-invasive, non-ionizing, and significantly cheaper and more versatile than CMR or CT techniques (18). Recently, several studies have proven that standard CMR techniques can quantify MR, mainly by calculating the difference between left ventricular stroke volume and aortic forward volume. MR quantified by this CMR method is better associated with patient outcomes than transthoracic echocardiography (TTE) (3). However, even standard CMR has issues in quantifying MR which could be circumvented by using 4D flow CMR. Three studies included in this systematic review have compared both 2-dimensional (2D)/3-dimensional (3D) echocardiography to 4D flow CMR methods of MR quantification.

Marsan et al. demonstrated that 2D echocardiography systematically under-estimated MR when compared to RT3DE. Importantly, RT3DE was comparable to 4D flow method. However, the RT3DE modality examined by Marsan et al. has its own limitations when observing MR and quantifying the regurgitant volume. Firstly, the interobserver reliability of 3D echocardiography is low for novice users (*R* = 0.51). Extended training and practice are therefore required to achieve reliable quantification of RT3DE measurements (19). There are also practical limitations of this technique; narrow-angle imaging is the most suitable for observing mitral valve anatomy, however, with this view the entire valve is not viewable in one live 3D echocardiography slice. RT3DE is therefore limited by the stitching artifacts of these slices (20). These artifacts are often exaggerated in patients with arrhythmias. In addition, the temporal resolution of RT3DE is reduced, offering ~25% the frame rate of its 2D counterpart. This temporal resolution makes scallop pathologies particularly difficult to detect through this method and other imaging modalities may need to be relied upon. In RT3DE several cardiac cycles are often needed to gain a full analysis of the valve, unlike the 2D echo (21). Some of these issues are applicable to 4D flow CMR methods as well and hence future studies need to consider investigating head-on-comparison between these methods in patients with arrythmias, especially atrial fibrillation or even high ectopic burden.

Another major limitation of 2D echocardiography highlighted by Gorodisky et al. is the geometric assumptions that must be made to quantify MR. The regurgitant jet eccentricity found in this study disproves the assumption made by color Doppler that PISA shape is hemispheric. In contrast to 2D color Doppler, 4D flow takes readings throughout systole and does not assume the area of regurgitant jet, resulting in a more reliable and accurate measurement of regurgitation volume.

**Figure 5:**
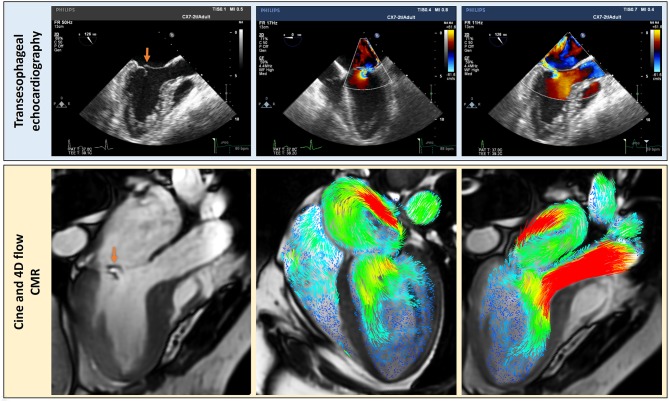
Complementary clinically relevant details can be assessed by using echocardiography; transesophageal echocardiography in this case (blue panel) and CMR with 4D flow streamline visualization. The MR flow convergence can be better visualized by echocardiography but 4D flow CMR is better to visualize MR jet within the left atrium. Hence, even though traditional methods of PISA and vena contracta have been proposed for 4D flow CMR, the current best method appears to be the direct MR jet quantification in the left atrium using the retrospective valve tracking method.

Transesophageal echocardiography (TOE) offers high-temporal and spatial resolution in the assessment of MR. This technique overcomes some of the above-mentioned problems with TTE, namely, lower spatial resolution and limited views. Shanks et al. compared both 2D and 3D TOE to CMR in 30 patients with MR. The standard method for quantifying MR with CMR (LVSV-AoPC) was used. For 3D TOE the regurgitant volume was calculated by measuring the mitral effective regurgitant orifice area and multiplying this by the velocity time integral of the regurgitant jet. The results of this study showed that 2D TOE again underestimated the regurgitant volume by 21.6 and 21.3% when compared to 3D TOE and CMR, respectively. Whereas, 3D TOE demonstrated remarkable agreement both in regurgitant volume and MR severity grading with CMR (22). Several studies have shown that 4D flow is comparable, if not better than, standard CMR. Therefore, it is plausible to conclude that 4D flow methods are comparable to 3D TOE (Figure 5), without the need for a semi-invasive procedure. In routine clinical practice, 4D flow MRI may be used in patients where TOE is not suitable as an alternative imaging modality (23).

The above-mentioned studies collectively demonstrate the 4D flow quantification methods are comparable to 3D echocardiographic techniques. With the developments in 4D flow MRI, seen in these papers, methods of accurate regurgitation volume quantification using CMR have the potential to be surpass 3D echocardiography.

### Comparison Studies to 2D PC MRI

Two-dimensional PC MRI is often the standard against which 4D flow CMR is compared. It is an established technique that clinically is used for blood flow assessment. Studies from Leiden have demonstrated that due to through-plane motion during acquisition, 2D PC tends to overestimate transmitral flow. This issue is circumvented by using 4D flow MRI which allows to track the valve plane to generate a dynamic PC plane through the mitral valve. Importantly, it also allows to track the mitral jet/jets during systole much more precisely.

It is worth noting that although echocardiography methods have advanced, some limitations remain, in particular the fundamental need to be parallel to the ultrasound beam. The Doppler-derived method for MR quantification used in Gorodisky's study typically examines a single frame in systole, often resulting in a large overestimation of the true regurgitation volume. The novel three-directional, cross-sectional velocity CMR-PISA method of quantification proposed in this study aims to overcome several limitations of other CMR techniques; including relying on accurate chamber volumes, flow calculations or assuming all other valves are healthy and that there is no intracardiac shunt.

Even though Feneis et al. demonstrated that 2D PC MRI is comparable to 4D flow for MR quantification, the authors commented that MR jets have the potential to change shape and direction significantly during systole especially with primary MR. This will impair measurements taken within a static imaging plane as in 2D PC MRI. It was also commented that 4D flow allows for both direct and indirect quantification without increasing scan time or requiring physician supervision.

Hence, the current evidence strongly suggests that valve tracking should be the preferred method for significant through-plane motion while quantifying MR directly.

### Automated Valve Tracking

A major drawback to the use of 4D flow MRI for MR quantification is the time-consuming process of manually tracking the mitral valve in every frame throughout the cardiac cycle. Automated valve tracking would streamline this process, decreasing analysis time (16) and improve both intra and inter-observer variability. However, what is noteworthy from Kamphuis et al.'s study, is that the automated valve tracking methods are not completely reliable for MR quantification. This is likely due to the fact that the MR jet or even jets do not necessarily follow the valvular plane and need independent tracking. This makes this procedure complex and further studies evaluating how this can be improved are warranted.

### Reproducibility and Reliability

Intraobserver and interobserver reliability of 4D flow was well-documented. The most reliable method seen in this systematic review was from Gorodisky et al.; whilst their 3D CMR-PISA method is not true 4D flow it did demonstrate excellent intraclass correlation (ICC = 0.99). Feneis et al. had the most comprehensive overview of 4D flow methods. Of the tested methods direct jet quantification with 4D flow was the most reliable (*r* = 0.929). Indirect quantification was slightly less reliable (*r* = 0.877) but both methods demonstrated high concordance with 2D PC for MR volume.

### Clinical Applications

**Figure 6:**
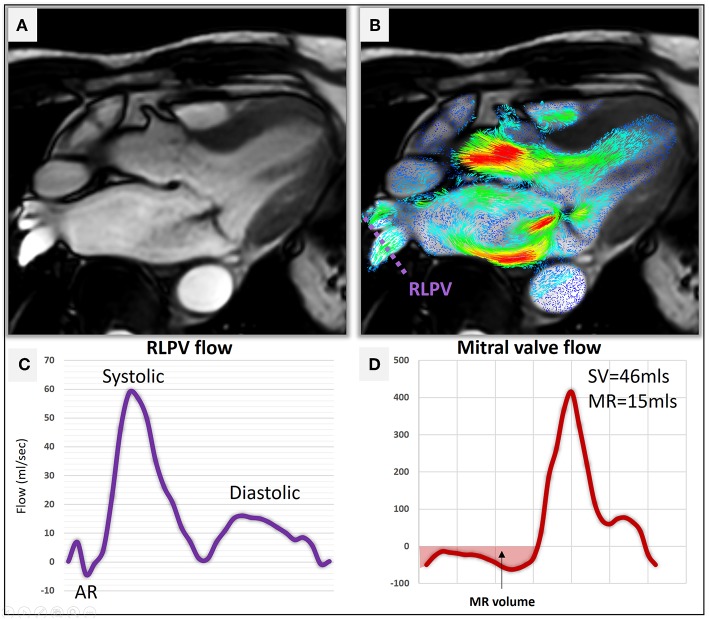
**(A)** A three-chamber cine demonstrating hypointense MR jet in the left atrium. **(B)** Two-dimensional velocity streamline overlay over the 3-chamber cine demonstrating flow acceleration of MR. A reconstructed plane (purple dotted line) was used to quantify pulmonary venous flow using 4D flow CMR. **(C)** Right lateral pulmonary venous (RLPV) flow demonstrates normal flow pattern (Systolic > Diastolic) with no systolic flow reversal. **(D)** The direct measure of MR volume using RVT was 15 mls suggestive of mild MR.

Current guidelines for the management of MR emphasize the severity of MR as a key criterion for clinical decision making (1). This review highlights that current literature supports the potential use of RVT method to quantify MR (Figure 6). Other methods including the 4D flow derived PISA methods demonstrate promising results.

With the advent of accelerated and free-breathing acquisitions for 4D flow, total scan times have significantly dropped to 8–10 min. In addition, there are now medical software packages offering RVT or direct flow jet analysis on 4D flow CMR data (*CAAS, Pie Medical Imaging, Maastricht, The Netherlands and Arterys Cardio, Arterys, San Francisco, CA*). This may allow rapid translation of this technology to benefit the patients. Significant reduction in analysis times through the use of automated valve tracking without impairing reliability will aid this translation into clinical practice. In the near future, 4D flow CMR is not likely to replace 2D TTE for MR assessment as TTE remains a very versatile and cheap investigation. However, in patients where further clarification of MR is needed, 4D flow CMR can provide reliable quantification of MR when compared with 3D TOE. The direct advantages of using 4D flow CMR over TOE are: it is completely non-invasive, does not require sedation and hence is a much more tolerable and safer test for the patients. Additionally, it can be used in patients with comorbidities that prevent the use of TOE (23). However, 4D flow MRI is not suitable in all cases and should therefore be used in a complementary nature to echocardiographic methods. Table 2 is a summarizing the advantages and disadvantages of the 4 imaging modalities discussed in this review. There are advantages to both 4D flow MRI and echocardiographic modalities for the assessment of MR which confirms that the two methods should be used to complement each other in clinical practice.

**Table 2:** The strengths and weaknesses of various non-invasive imaging modalities in the clinical assessment of the mitral valve and MR.

	**TTE**	**TOE**	**sCMR**	**sCMR + 4D Flow**
Etiology of MR	++	+++	+	++
Carpentier classification	++	+++	+	+
Number of regurgitant Jets	++	++	++	+++
Eccentricity of jet	++	++	++	+++
LV volume/function	+	++	+++	+++
LA volume	+	+	+++	+++
LV scar	+	+	+++	+++
LV edema	–	–	+++	+++
MR volume				
Direct	–	–	+	+++
Indirect	+	++	+++	+++

*4D flow CMR is complementary to TTE, TOE, and standard CMR (sCMR). The addition of 4D flow to sCMR allows for better direct quantification of MR and visualization of regurgitant jets*.

### Limitations of 4D Flow

There are many challenges facing adoption of 4D flow CMR. The current 4D flow CMR workflow requires extensive training and experience. Till lately, there were no robust software tools available to quantify flow using 4D flow datasets. Even though this is changing, the software tools developed by major vendors need further advancement to make the analysis more intuitive and simple. One major problem we face is in the development of understanding of the true “three-dimensional” velocity encoding visualization vs. one-directional velocity encoding imaging (echocardiography) which we are so used to. In addition, current technology prohibits live visualization of 4D flow data as it requires reconstruction and the hardware vendors do not have any software packages capable of further analysis. However, a quick quality check of the data can still be done slice by slice on each directional dataset on the workstation. This includes looking for any breathing, pixilation and aliasing artifacts in the region of interest. Repeating 4D flow CMR can be a time-consuming process and hence usually the optimum velocity (VENC) should be determined using 2D PC acquisition prior to planning cross-sectional 4D flow CMR. As 4D flow CMR is essentially a PC acquisition, it can be used for flow quantification in clinical practice. However, clinical outcome data demonstrating its non-inferiority to current method are still warranted.

### Future Work

**Figure 7:**
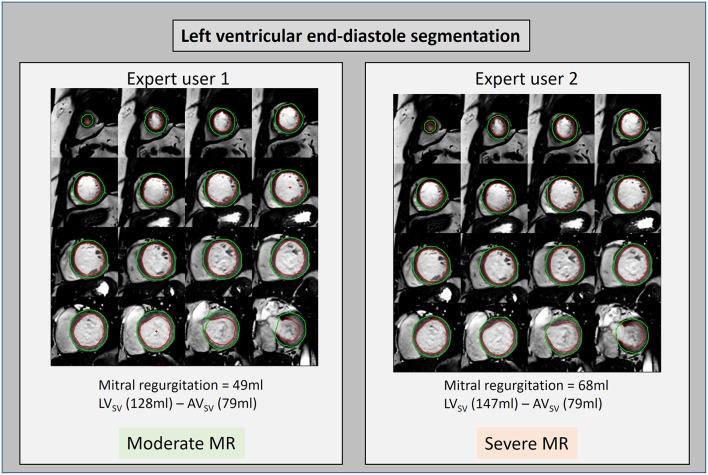
LV volume contours drawn by two different expert users on short-axis cine stack. The left ventricular stroke volume was calculated by both users and found to be 128 and 147 ml, respectively. This difference in calculated stroke volume led to a discrepancy in the classification of this MR case potentially affecting patient outcomes.

The three-directional, 3D, cross-sectional velocity data acquired by 4D flow CMR allows quantification of flow and regurgitation in many novel ways. These methods could include particle tracing component analysis, intra-cavity energetics (24–26) RVT to quantify forward and regurgitant jet, 4D flow derived PISA, semi-quantitative streamline visualization and intra-cavity hemodynamic forces. The relevance of these methods needs to be tested and investigated to inform the severity of MR, left ventricular hemodynamics and predict adverse remodeling. In addition, it remains unclear which method of MR quantification is more reliable. Even though the current standard method to quantify MR by CMR has demonstrated clinical outcome benefit over echocardiography, its reliability is still debated as slight changes in contouring LV volumes can result in significant different MR volume quantification (Figure 7). Future studies need to prospectively evaluate the clinical outcome benefit of using 4D flow CMR for MR quantification. In cases of multiple jets of MR, even RVT segmentation of individual jets, in separate planes, can be a time-consuming and challenging task. Hence, novel semi-quantitative and quantitative 4D flow methods are needed which can simplify the process of MR quantification without compromising on precision. One promising 4D flow MRI technique is particle tracing. This requires generating synthetic particles from the 4D MRI images and tracking these over the cardiac cycle. The path of these particles can be visualized as a movie or as a static image of the particle path. It is yet to be seen if particle tracing can offer benefit other 4D flow MR quantification methods (27).

## Conclusion

The evidence gathered in this systematic review suggests that 4D flow derived MR quantification methods are reliable and circumvent issues with standard imaging. These methods, namely RVT, offer an alternative imaging modality to quantify MR in patients with limited acoustic window on TTE or who need further clarification on the severity of MR. There is a growing body of evidence demonstrating that 4D flow derived MR quantification is as accurate as quantified by 3D semi-invasive TOE. Further studies are warranted to demonstrate if 4D flow CMR derived MR quantification offers improved predictability for clinical outcomes over standard imaging.

## Data Availability

All datasets for this study are included in the manuscript and the supplementary files.

## Author Contributions

PG and AS conceived the idea and need for the systematic review. BF, NBar, and GA prospectively registered the systematic review. BF, PG, and AS did the literature search. BF, NBar, NBal, PG, AR, and AS drafted the initial version. GA, RH, NBri, and LO'T provided multi-parametric imaging data as part of the EurValve study (http://www.eurvalve.eu). JR, RvdG, RH, and JW provided expert critical input to the content independently. AA-M and GF provided critical input into clinical applications of the emerging CMR techniques.

### Conflict of Interest Statement

The authors declare that the research was conducted in the absence of any commercial or financial relationships that could be construed as a potential conflict of interest.
